# Loss of enhancer of zeste homologue 2 (EZH2) at tumor invasion front is correlated with higher aggressiveness in colorectal cancer cells

**DOI:** 10.1007/s00432-019-02977-1

**Published:** 2019-07-17

**Authors:** Julian Böhm, Julienne Kathrin Muenzner, Aylin Caliskan, Benardina Ndreshkjana, Katharina Erlenbach-Wünsch, Susanne Merkel, Roland Croner, Tilman T. Rau, Carol Immanuel Geppert, Arndt Hartmann, Adriana Vial Roehe, Regine Schneider-Stock

**Affiliations:** 1 Experimental Tumorpathology, Institute of Pathology, Friedrich-Alexander-University of Erlangen-Nürnberg, Universitätsstr. 22, 91054 Erlangen, Germany; 2 Institute of Pathology, Friedrich-Alexander-University of Erlangen-Nürnberg, Krankenhausstr. 8-10, 91054 Erlangen, Germany; 3 Department of Surgery, Friedrich-Alexander-University of Erlangen-Nürnberg, Krankenhausstr. 12, 91054 Erlangen, Germany; 4 Department of Surgery, Otto-von-Guericke University, Leipziger Str. 44, 39120 Magdeburg, Germany; 5 Institute of Pathology, University Bern, Murtenstr. 31, 3008 Bern, Switzerland; 6 Department of Pathology, Federal University of Health Sciences of Porto Alegre (UFCSPA), R. Sarmento Leite, 245-Centro Histórico, Porto Alegre, RS 90050-170 Brazil

**Keywords:** EZH2, EZH2 inhibition, Colorectal cancer, Tumor invasion front, Worse prognosis, CAM assay

## Abstract

**Purpose:**

Enhancer of zeste homolog 2 (EZH2) is associated with epigenetic gene silencing and aggressiveness in many tumor types. However, the prognostic impact of high EZH2 expression is controversially discussed for colorectal cancer. For this reason, we immunohistochemically analyzed EZH2 expression in 105 specimens from colon cancer patients separately for tumor center and invasion front.

**Methods:**

All sections from tissue microarrays were evaluated manually and digitally using Definiens Tissue Studio software (TSS). To mirror-image the EZH2 status at the tumor invasion front, we treated HCT116 colon cancer cells with the EZH2 inhibitor 3-Deazaneplanocin A (DZNep) and studied the growth of in ovo xenografts in the chorioallantoic membrane (CAM) assay.

**Results:**

We showed a significant decrease in EZH2 expression and the repressive H3K27me3 code at the tumor invasion front as supported by the TSS-constructed heatmaps. Loss of EZH2 at tumor invasion front, but not in tumor center was correlated with unfavorable prognosis and more advanced tumor stages. The observed cell cycle arrest in vitro and in vivo was associated with higher tumor aggressiveness. Xenografts formed by DZNep-treated HCT116 cells showed loosely packed tumor masses, infiltrative growth into the CAM, and high vessel density.

**Conclusion:**

The differences in EZH2 expression between tumor center and invasion front as well as different scoring and cutoff values can most likely explain controversial literature data concerning the prognostic value of EZH2. Epigenetic therapies using EZH2 inhibitors have to be carefully evaluated for each specific tumor type, since alterations in cell differentiation might lead to unfavorable results.

**Electronic supplementary material:**

The online version of this article (10.1007/s00432-019-02977-1) contains supplementary material, which is available to authorized users.

## Introduction

As the catalytic subunit of the polycomb repressive complex (PRC2), histone methyltransferase EZH2, preferentially methylates lysine 27 on histone 3 (H3K27) and is associated with epigenetic gene silencing (Simon and Lange 2008; Kuzmichev et al. 2002; Margueron and Reinberg 2011). Besides its functional role for cell cycle regulation, EZH2 is also involved in metastasis by modulating tumor angiogenesis and the epithelial-to-mesenchymal transition, a process by which disseminating tumor cells acquire mesenchymal characteristics to migrate through the extracellular matrix (Simon and Lange 2008; Kim and Roberts 2016; Crea et al. 2012). In different studies, overexpression of EZH2 has been identified as a biomarker of aggressive and highly proliferating tumors and is, hence, being associated with worse patient outcome, e.g., in endometrial, prostate, and breast cancer as well as melanoma (Bachmann et al. 2006). In colorectal cancer (CRC), EZH2 expression could also be linked to cancer stem cell potential (Chen et al. 2016). Fussbroich et al. investigated the expression of EZH2 in colon cancer and adenomas immunohistochemically and reported a significant increase in EZH2 expression in CRC, especially in less differentiated carcinomas (Fussbroich et al. 2011). Even though there is a general agreement about an increase in EZH2 expression in CRC compared to normal tissue, its prognostic value is controversially discussed (Benard et al. 2014; Fluge et al. 2009; Liu et al. 2015; He et al. 2014). While some studies report that CRC patients having a high expression of EZH2 show a lower overall disease-free survival (DFS) (Liu et al. 2015; He et al. 2014; Mimori et al. 2005), other reports describe that a high expression of EZH2 in CRC patients is correlated with an improved relapse-free survival (Benard et al. 2014; Fluge et al. 2009; Takawa et al. 2011). In a very recent report by Yamamoto et al., a significant association between low EZH2 expression and shorter progression-free survival in 64 CRC patients was shown (Yamamoto et al. 2017). Interestingly, there is a stronger agreement concerning the significance of the EZH2-dependent H3K27me3 code for cancer prognosis. In most studies, hypomethylated H3K27 was shown to be correlated with tumor aggressiveness in breast, ovarian, and pancreatic cancer as well as in colon carcinoma (Tamagawa et al. 2013; Wei et al. 2008). Hence, there might be cancer-related EZH2 functions that are independent from H3K27 methylation. With respect to the analysis of EZH2 expression in CRC, the use of various scoring systems, different cutoff values, or mixed patient cohorts of colon and rectal tumors make it very difficult to compare the different studies and question the clinical application of EZH2 as a reliable biomarker. So far, there are also no studies considering a possible heterogeneity of EZH2 expression in the different tumor areas such as tumor center and invasion front.

In this study, we analyzed the expression of EZH2 in a cohort of 105 colon cancer patients with a median follow-up of 99.7 months. In particular, we were interested in the role of EZH2 in certain tumor areas. Thus, we focused on the invasion front, where cancer cells disseminate and migrate into the surrounding tissue. We propose a possible explanation for the strongly opposing data in the literature in terms of EZH2 expression levels and their prognostic value in CRC. Moreover, for the first time we suggest a possible link between cell cycle stop and higher aggressiveness when EZH2 is inhibited, a phenomenon that is well known for the tumor invasion front. Our semi-automated software workflow can be applied for an objective and time-efficient analysis of immunohistochemical nuclear staining signals.

## Materials and methods

### Patient and tissue selection

We retrospectively analyzed a tumor group of 268 cases with primary adenocarcinoma of the colon that has not received neoadjuvant therapy and has undergone surgical resection at the University Hospital Erlangen (FAU Erlangen-Nürnberg) between 2005 and 2009. Clinical information and follow-up data were collected prospectively by the Erlangen Registry for Colorectal Carcinoma (ERCRC). This retrospective study is covered by ethic votes of the FAU (24.01.2005, 18.01.2012). For clinical correlation of the data, we defined that the complete clinical data set and at least two tissue samples per patient both have to be available, at the invasion front and in the center, to avoid bias caused by tumor heterogeneity. This led to a final number of 105 patients. This cohort included 54 (51.4%) men and 51 (48.6%) women. Median of the patient’s age was 66 years (range 39–89). 56 (53.3%) of the patients had an UICC stage of I or II and 49 (46.7%) were staged as III or IV. While 80 (76.2%) patients did not carry distant metastases and 25 (23.8%) patients had M1 tumors, 45 (42.9%) patients showed lymph node metastases. The median follow-up time was 99.7 months (range 1–136 months). The 5-year cancer-related survival rate was 75.4%; at the time of analysis, 55 of the 105 patients (52.4%) had died, 33 (31.4%) died of disease (Table 1). Tumor budding has been scored according to the guidelines of the International Tumor Budding Consensus Conference 2016 (Lugli et al. 2017). Examples are given in Online Resource 1.

**Table 1:** Patient characteristics and association with immunohistochemical EZH2 expression

Feature	Frequency *N* (%)	EZH2-score IF ≤ 30 (%)	EZH2 score IF > 30 (%)	*p* value
Gender				
Male	54 (51.4)	30 (55.6)	24 (44.4)	ns
Female	51 (48.6)	24 (47.1)	27 (52.9)	
Patient age				
Median (min, max)	66 (39–89)	–	–	ns^a^
Histological subtype				
Adenocarcinoma (NOS)	88 (83.8)	44 (50.0)	44 (50.0)	
Mucinous	13 (12.4)	7 (53.8)	6 (46.2)	
Other	4 (3.8)	3 (75.0)	1 (25.0)	ns
UICC				
I	20 (19.1)	7 (35)	13 (65)	
II	36 (34.3)	14 (38.9)	22 (61.1)	
III	24 (22.8)	13 (54.2)	11 (45.8)	
IV	25 (23.8)	20 (80)	5 (20)	0.005**
Grading				
1	5 (4.8)	1 (20.0)	4 (80.0)	
2	63 (60.0)	32 (50.8)	31 (49.2)	
3	37 (35.2)	21 (56.8)	16 (43.2)	ns
pT				
pT1	6 (5.7)	2 (33.3)	4 (66.7)	
pT2	17 (16.2)	8 (47.1)	9 (52.9)	
pT3	60 (57.1)	29 (48.3)	31 (51.7)	
pT4	22 (21.0)	15 (68.2)	7 (31.8)	ns
cM/pM classification				
M0	80 (76.2)	34 (42.5)	46 (57.5)	
M1	25 (23.8)	20 (80.0)	5 (20.0)	0.01*
pN classification				
pN0	60 (57.1)	24 (40.0)	36 (60.0)	
pN1–2	45 (42.9)	30 (66.7)	15 (33.3)	0.004**
Localization				
Cecum	17 (16.2)	8 (47.1)	9 (52.9)	
Ascending colon	26 (24.8)	14 (53.8)	12 (46.2)	
Transverse colon and flexures	15 (14.3)	7 (46.7)	8 (53.3)	
Descending colon	6 (5.7)	2 (33.3)	4 (66.7)	
Sigmoid colon	41 (39.0)	23 (56.1)	18 (43.9)	ns
Localization^b^				
Proximal	54 (51.4)	27 (50)	27 (50)	
Distal	51 (48.6)	27 (52.9)	24 (47.1)	ns
Perineural invasion				
Yes	7 (6.7)	4 (57.1)	3 (42.9)	
No	96 (91.4)	49 (51)	47 (49)	
Unknown	2 (1.9)	1 (50)	1 (50)	ns
Lymphatic invasion				
Yes	28 (26.7)	16 (57.1)	12 (42.9)	
No	77 (73.3)	38 (49.4)	39 (50.6)	ns
MMRP expression				
Loss^c^	25 (23.8)	13 (52)	12 (48)	
Intact	80 (76.5)	41 (51.2)	39 (48.8)	ns
Tumor budding				
Low	56 (53.3)	24 (42.9)	32 (57.1)	
Intermediate	28 (26.7)	18 (64.3)	10 (35.7)	
High	21 (20)	12 (57.1)	9 (42.9)	ns

*IF* invasion front, *ns* non-significant

*p < 0.05, **p < 0.01

^a^Pearson correlation coefficient = − 0.068 (*p* = 0.489)

^b^Proximal: cecum, ascending, right flexure, transverse; distal: left flexure, descending, sigmoid

^c^Loss of MMRP expression in at least one of the four markers hMLH1, hMLH2, hMSH6, and PMS2

Specimens were formalin fixed and paraffin embedded (FFPE). Histopathological review of all diagnostic cases was performed by three pathologists (TTR, CG, AH). The tumors were staged according to the eighth ed. of the UICC TNM classification.

### TMA construction

TMA has been constructed as previously described (Nolte et al. 2017). For this, three representative punches with a diameter of 0.6 mm were taken out of both the tumor center and the invasion front for each patient using a TMA Grand Master (3DHISTECH, Budapest, Hungary; Online Resource 2) and then cut to a thickness of 3 µm.

### Cell lines

The human colorectal tumor cell line HCT116 (CCL-247, ATCC) was kept in RPMI medium supplemented with 10% FBS and 1% penicillin/streptomycin. Cells were cultured in a humidified incubator at 37 °C with 5% CO_2_.

The cell line was authenticated using Multiplex Cell Authentication by Multiplexion (Heidelberg, Germany). Mycoplasma-free status has been verified.

### Crystal violet assay

HCT116 cells were seeded in 96-well plates (10,000 cells/well) and incubated for 24 h. Cells were then treated with a concentration range of 3-Deazaneplanocin A (DZNep, 250 nM to 100 µM) for another 48 h. Afterward, cells were washed with 200 µL of PBS and stained with 50 µL of a Crystal Violet staining solution (0.5% Crystal Violet; 20% MeOH in ddH_2_O) for 15 min at room temperature. Finally, cells were washed three times with 200 µL of ddH_2_O and the plates were air dried before dissolving the Crystal Violet in 10% acetic acid (200 µL). Absorption was measured at 595 nm using a Victor™ X3 2030 Multilabel Plate Reader (PerkinElmer). Percentages of viable cells were calculated compared to respective DMSO controls (100% of viable cells).

### Western blot analysis

HCT116 cells (1.5 × 10^6^ cells) were seeded into 10-cm cell culture dishes and incubated for 24 h. Then cells were treated with DZNep (5 µM) for 1, 3, 6, 24, and 48 h. Untreated HCT116 cells were served as control. After the indicated incubation periods, cells were harvested, washed in PBS, and proteins were extracted by lysis with Urea Lysis Buffer. Protein concentration was measured using the Bio-Rad DC™ Protein Assay (BioRad). Equal amounts of lysates were applied to SDS-PAGE and proteins were then transferred to a nitrocellulose membrane (Amersham Protran Premium 0.2 NC, GE Healthcare Life Sciences) prior to probing with antibodies. Antibodies were applied as follows: anti-EZH2 (1:10,000, EZH2 (D2C9) XP, monoclonal rabbit, Cell Signaling), anti-H3K27me3 (1:10,000, Tri-Methyl-Histone H3 (Lys27) (C36B11, monoclonal rabbit, Cell Signaling), anti-GAPDH-HRP as loading control (1:50,000, clone 6C5, monoclonal mouse, Abnova), and secondary HRP-conjugated antibody (1:10000, Goat anti-Rabbit IgG (H + L, Thermo Scientific). Signal of protein bands was detected using chemiluminescent HRP substrate (Immobilon™ Western Chemiluminescent HRP substrate, Millipore) according to the manufacturer’s instructions and the GeneGnome detection system (Syngene).

### CAM assay

To study the in vivo growth pattern of tumor cells with decreased EZH2 activity, we treated HCT116 cells (1.5 × 10^6^) with the EZH2 inhibitor DZNep (5 µM, IC_30_ range as determined by Crystal Violet and applied in the literature) for 48 h (Sha et al. 2015). The in ovo CAM assay has been performed as described previously (Ribatti 2017; Muenzner et al. 2018). Specific pathogen-free (SPF) eggs were bred in an incubator at 37 °C and in a relative humidity of 70–80%. On day 8 of chicken embryo development, a window (Ø ~ 1–1.5 cm) was cut into the more rounded pole of the eggs and the egg shell membrane was removed. The windows were sealed with silk tape (Durapore™, 3 M) and the eggs were incubated for another day. Then control cells (untreated, *n* = 10) and DZNep-treated (*n* = 9) HCT116 cells (1 × 10^6^) were embedded in Matrigel (Corning^®^ Matrigel^®^ Basement Membrane Matrix, 356237; 1:1 mixture with medium; total volume 40 µL per pellet) and the resulting pellets were placed on the CAM of the developing embryos (day 9 of incubation). Microtumors with their surrounding CAM were harvested 5 days after engraftment on the CAM, fixed in 4% phosphate buffered formalin for 24 h, and embedded in paraffin. The tumor volume was calculated using a formula for ellipse calculation prior to fixation: Tumor volume = pellet length*width*height* × 0.52.

### Cell cycle analysis

To verify the known G1-arresting effects of DZNep, 1.25 × 10^6^ cells were seeded into 6-cm cell culture dishes and incubated for 24 h (Sha et al. 2015). Then cells were treated with 5 µM DZNep or a respective amount of DMSO (solvent control) for an additional 24 h. Finally, cells were harvested by trypsinization, washed with PBS, fixed in 70% ethanol overnight (4 °C), rehydrated and stained with propidium iodide (30 min at room temperature in the dark; propidium iodide staining buffer: 224 µM propidium iodide, 3.875 mM sodium citrate, 0.1% Triton X-100, and 0.5 mg/mL RNase A in PBS,). Cell cycle analysis was performed using a FACS Calibur flow cytometer (BD). Per sample 10,000 single cells were analyzed and the different cell populations (sub-G1 fraction, G1-, S- and G2/M-phase) were determined using the FlowJo software (FlowJo7.6.5).

### Immunohistochemistry of CAM tumors

Serial sections (2 µm) were cut from the paraffin blocks and mounted on pre-coated slides for immunohistochemical analysis of the CAM tumors.

All FFPE whole (CAM) tissue sections and TMAs were deparaffinized with xylene and rehydrated with graded ethanol. The antigen retrieval was performed by 1 min steam cooking in TRS buffer pH 6. Slides were incubated at 4 °C overnight with primary monoclonal antibody EZH2 1:500 (Rabbit, D2C9, Cell Signaling) or anti-H3K27me3 1:300 (Rabbit, C36B11, Cell Signaling). Antibody binding was visualized using the Polymer-Kit (AP, Zytomed). Adjacent slides were stained with hematoxylin and eosin for histomorphological analyses. Immunohistochemical scoring was performed in a semi-quantitative way by two researchers (AVR, JB). Intensity was quantified by nuclear reaction in a range from 1 to 3 (Online Resource 3). Area was quantified by percentage of positive cells in 5% steps. EZH2 and H3K27me3 immunoscores were generated by multiplying intensity (0–3) with the respective percentage of positive cells (0–100%). The manual analysis only considered the groups of highest intensity for each punch. The EZH2 score was then used for correlation with clinical data. Samples were only included, if at least two of three punches were analyzable.

The tumor cell area of the CAM microtumors was measured using the Case Viewer software (3D HISTECH ltd. Version 2.0) and scans of HE-stained slides were performed using a digital slides scanner (Scanner FLASH II using CIS VCC FC60FR19CL Camera (0.19 microns/pixel in 40 ×), 3D-Histech, Budapest, Hungary). The tumor cell area was then calculated as a percentage of the total microtumor area including residual Matrigel but excluding the CAM tissue. For the quantification of blood vessels, we used HE-stained slides and included only blood vessels containing nucleated chicken erythrocytes. Besides counting the amount of blood vessels of the microtumors (intra- and peri-tumoral), the relative vessel density was determined by dividing the area of blood vessels by the total microtumor area including residual Matrigel.

### Immunohistochemistry of mismatch repair proteins

Immunohistochemistry was performed on 2-μm-thick sections of FFPE tumor blocks on a Ventana BenchMark Ultra automated instrument (Ventana Medical Systems, Inc., Tucson, AZ, USA) according to routine standards of our institute and manufacturer’s recommendations. The following mouse monoclonal antibodies have been used: MLH1 (ES05, 1:50, DAKO), MSH2 (clone 760-4265, ready-to-use, Ventana), MSH6 (clone 44/MSH6, 1:100, BD), PMS2 (clone EP51, 1:40, DAKO).

Heat-induced epitope retrieval was performed using CC1 at 95 °C for 36 min for tissue preparation for each staining. Then incubation of the pre-treated tissue sections was followed for 20 min at room temperature for the MLH1 antibody, whereas for the MSH2 antibody incubation at 37 °C for 16 min and for the MSH6 and PMS2 antibody 30 min at room temperature was performed. Binding of the antibodies to the antigen was visualized using the ultraView Universal DAB Detection Kit, and subsequently, sections were counterstained with Hematoxylin and Bluing Reagent (Ventana). Assessment of MLH1, MSH6, MSH2, and PMS2 was performed by two independent observers (CG, AH) who were blinded to the clinical data. For all four IHC stainings, a nuclear staining of > 10% of the tumor cells was considered as a positive expression. The absence of staining at > 90% of all tumor cells was considered as loss of MMRP but positive internal control was checked (e.g., endothelial cells or cells of the connective tissue). Analysis has been performed according to the German S3 guidelines for colon cancer in its actual version (German S3-Guidelines Colorectal Cancer 2019).

### Computer-assisted digital and manual analysis

Stained slides were scanned using a Pannoramic MIDI (Camera type: CIS_VCC_F52U25CL, Objective name: Plan-Apochromat, Objective magnification: 40 ×, Camera adapter magnification: 1 ×) (3DHISTECH, Budapest, Hungary). Pannoramic Viewer software Version 1.15.4 (3DHISTECH, Budapest, Hungary) was used to view and annotate all TMAs and whole slides. We applied Definiens Tissue Studio Software Version 4.0 (TSS; Definiens AG, Munich, Germany) to obtain a semi-automated score for all cores on the center TMAs and correlated it to the same score, which was acquired by manual analysis. This TSS score was generated by multiplying intensity (0–3) with percentage of the positive cells (0–100%) resulting in values of 0–300. In contrast to manual analysis, the TSS analyzed all staining intensities.

For the correlation of EZH2/H3K27me3 code scores derived by either the TSS software or the pathologist, we used all punches that were available for both stainings, independent of center and invasion front (268 patients × maximum 6 punches per case = 1608). Not all punches were analyzable by the pathologists or the TSS due to artifacts or a very low number of cells. Only punches were included when both stainings for EZH2 and H3K27me3 were available. This led to the final number of 766 different punches for statistical evaluation of correlation.

**Fig. 1:**
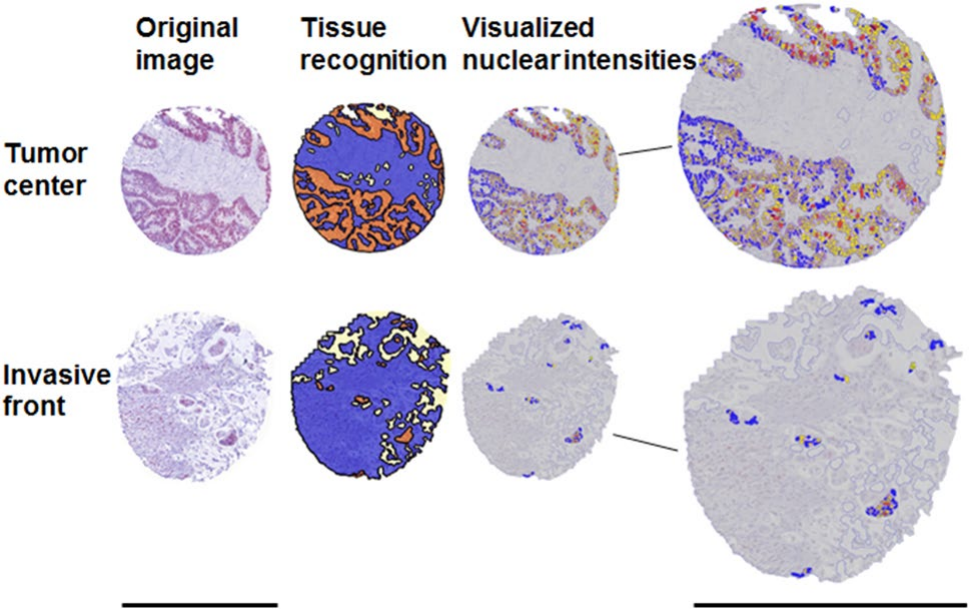
Image analysis of Tissue Studio at different steps. Original images with EZH2 staining are shown left. Tissue recognition demonstrates the marked ROIs (orange: cancer cells, blue: stroma, light yellow: white space). The same cores after usage of the Tissue Studio cellular analysis tool are shown right. Blue nuclei are negative for EZH2; yellow, orange, and red colors represent intensities 1, 2, and 3, respectively. Scale bar 600 µm

By manual training of the software and control of the regions of interest (ROIs, e.g., stroma or epithelial cells) that the software recognizes, we guaranteed a minimum of mistakes by the “Composer: Training” tool, which annotates the ROIs. The workflow contained marking ROIs by hand in as many examples and as accurate as possible. After this extensive training, the software was able to mark ROIs on its own. Nevertheless, we randomly chose software annotated slides to review whether the training was performed properly and corrected annotations if necessary. The same procedure was used for every step, in which the software had to select and classify different tissues or intensities. Different nuclear intensities were taught to the software using representative examples of those intensities (Fig. 1), and then adjusting the thresholds with TSS. Nuclei underneath 20% of the typical nucleus size measured by the software were excluded to reduce the impact of artifacts.

With the experience acquired during the semi-automated TMA analysis, we edited eight whole slides in an identical manner to establish heatmaps using the “Heatmap” feature, which is accessible via TSS, to clearly display our results in the tumor center and at the invasion front.

### Statistical analysis

The correlations between coded EZH2 score (cutoff score = 30 as median) and clinicopathological variables (sex, localization, subtype, pT-, pN-, and M-category, UICC stage, grading) were calculated using the *χ*^2^ test or Fisher’s exact test in cross tables. Unpaired *t* test was used to correlate EZH2 immunoscore and clinicopathological parameters. For direct comparison of the values at the invasion front and the tumor center, paired *t* tests (Wilcoxon) were applied. Spearman correlation was used to compare the EZH2 and H3K27me3 scores determined by the pathologists and the Definiens TSS. All tests were two sided. The Kaplan–Meier curves of cancer-related survival were compared using a log-rank test. Death from unrelated causes has been censored. Univariate Cox regression analysis was performed to evaluate the risk of dying of disease for EZH2 and clinicopathological parameters. All variables with *p* < 0.05 in univariate analysis were included into a multivariate model to identify independent prognostic factors. *p* values of < 0.05 were considered to be statistically significant. The statistical analysis was performed using SPSS Version 21 (IBM, Armonk, New York).

## Results

Selected clinical data of patients and EZH2 immunoscores are presented in Table 1 to give an overview of the 105 patients investigated.

### EZH2 and H3K27me3 stainings

**Fig. 2:**
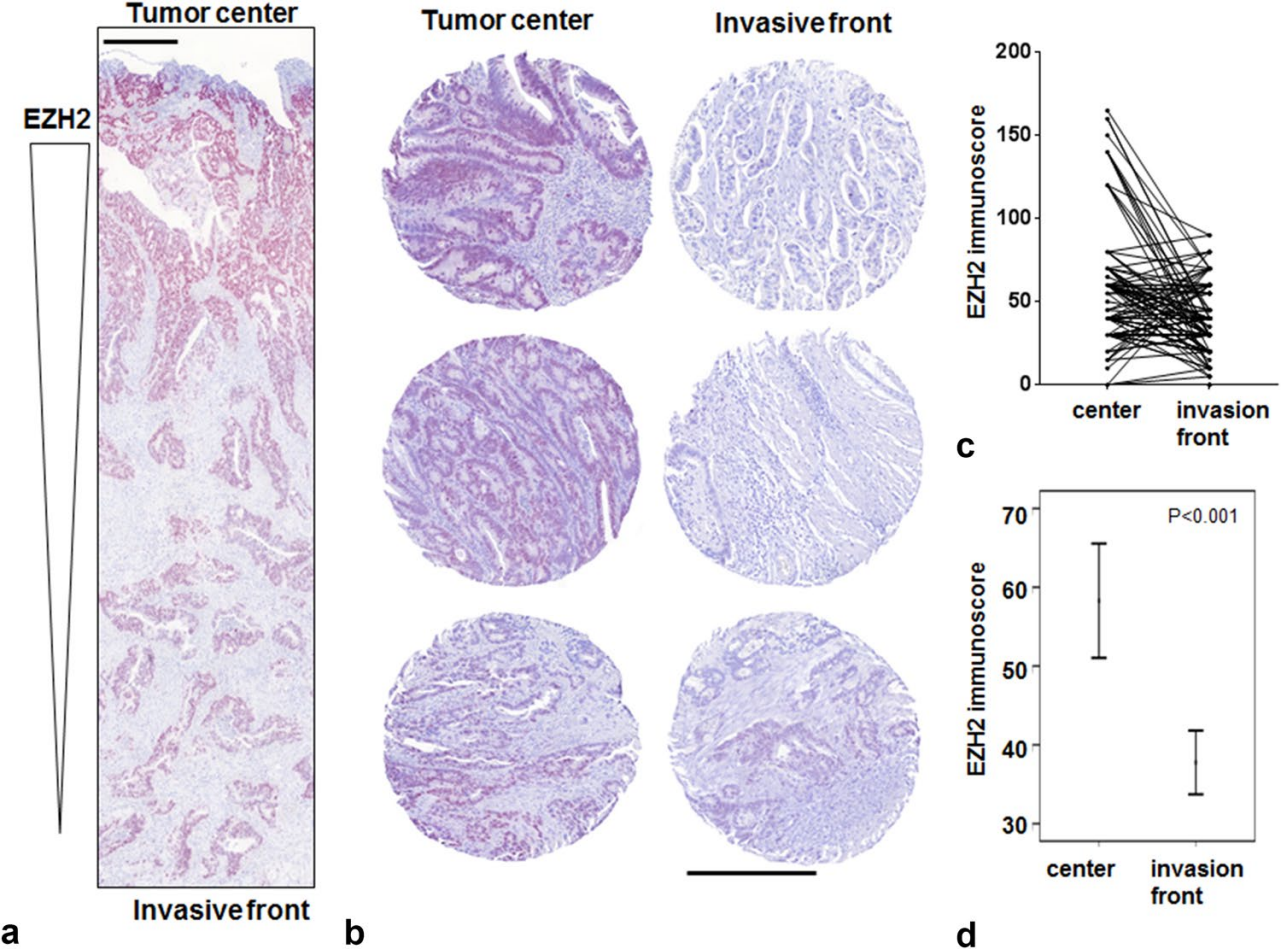
Comparison of EZH2 staining intensity in tumor center and at the invasion front. **a** EZH2 staining intensity decreases from tumor center to invasion front (× 4 magnification) considering a whole slide (**b**) and three TMA cases (× 10 magnification). Tumor center is shown on the left, invasion front on the right side. **c**, **d** Statistical analysis (line diagram, error bar diagram) clearly verifies a loss of EZH2 expression at the invasion front (*n* = 105; *p* < 0.001). Scale bar 400 µm

Both stainings were majorly found in the nucleus of the tumor cells (Fig. 2a, b) with a significant decrease in EZH2 expression at the tumor invasion front (*p* < 0.001, Fig. 2c, d). The EZH2 and the H3K27me3 stainings were almost homogeneous (Online Resource 2) demonstrating that the punches were representative of the respective whole tumor slices. Correspondingly, there was a significant decrease in the H3K27me3 code at the invasion front (*p* < 0.001). When evaluating the 766 punches for correlation between EZH2 and H3K27me3 code, we did not find a significant correlation between both markers.

### Clinicopathological data and survival analysis

We could confirm that MMRP loss occurs more frequently in the proximal colon (proximal: 16/54 (29.6%) and distal: 9/51 (17.6%). We identified low and intermediate tumor budding in 80% of cases whereas high tumor budding was found in 21 tumors (20%; Table 1).

Follow-up data were available for all 105 colon cancer patients. The cancer-related 5-year survival rate was 75.4% (95% CI 67.0–83.8). Males affected by colon cancer had a worse prognosis than females (Online Resource 4a). The Kaplan–Meier curve reflected the prognostic value of UICC stage (Online Resource 4b) with patients having a high UICC stage showing a worse cancer-related survival. pT_3_ and pT_4_ categories were significantly correlated with a worse prognosis (Online Resource 4c). The occurrence of lymph node metastases and distant metastases was significantly associated with a worse prognosis for patients (Online Resource 4d, e). High tumor budding was significantly correlated with worse prognosis (Online Resource 4f). The stratification regarding the tumor grade (Online Resource 4g), MMRP status, and proximal/distal localization did not reach significance (data not shown). Consistently, Univariate Cox analysis revealed a high prognostic value for M status, pN category, pT category, and tumor budding with significant *p* values < 0.05 (Table 2). When including all parameters that were significant in the Univariate Cox analysis, distant metastasis and EZH2 difference were confirmed as independent prognostic factors (Table 2).

**Table 2:** Prognostic significance (cancer-related survival) of clinicopathological parameters using the Cox’s regression model

Parameter	*N*	Cancer-related survival		
		Relative risk	95% confidence interval	*p* value
*Univariate analysis*				
pT category	105	12.3	1.7–90.3	0.013*
pN category	105	5.5	2.5–11.8	< 0.001***
M category	105	18.8	8.4–42.1	< 0.001***
EZH2 score 30	105	0.4	0.2–0.9	0.027*
EZH2 difference^a^	105	2.1	1.0–4.2	0.042*
Tumor grading	105	1.3	0.7–2.7	0.403
Sex	105	0.5	0.2–1.0	0.064
Age (66 cutoff)	105	1.3	0.6–2.5	0.488
Localization^b^	105	1.3	0.7–2.6	0.438
MMRP loss	105	1.0	0.4–2.2	0.948
Tumor budding^c^	105	2.5	1.2–5.3	0.016*
*Multivariate analysis*				
pT category	105	4.2	0.5–33.3	0.173
pN category	105	2.1	0.9–5.2	0.091
M category	105	12.3	4.8–31.6	< 0.001***
EZH2 score 30	105	1.6	0.6–3.9	0.318
EZH2 difference^a^	105	2.8	1.3–6.2	0.012*
Tumor budding^c^	105	1.4	0.6–3.4	0.404

*p < 0.05, ***p < 0.001

^a^EZH2 score (Δtumor center–tumor invasion front)

^b^Proximal versus distal

^c^Low/intermediate versus high

Our findings demonstrate that the sampling was accurate and our tumor group is representative for colon cancer.

### EZH2 expression and clinical outcome

**Fig. 3:**
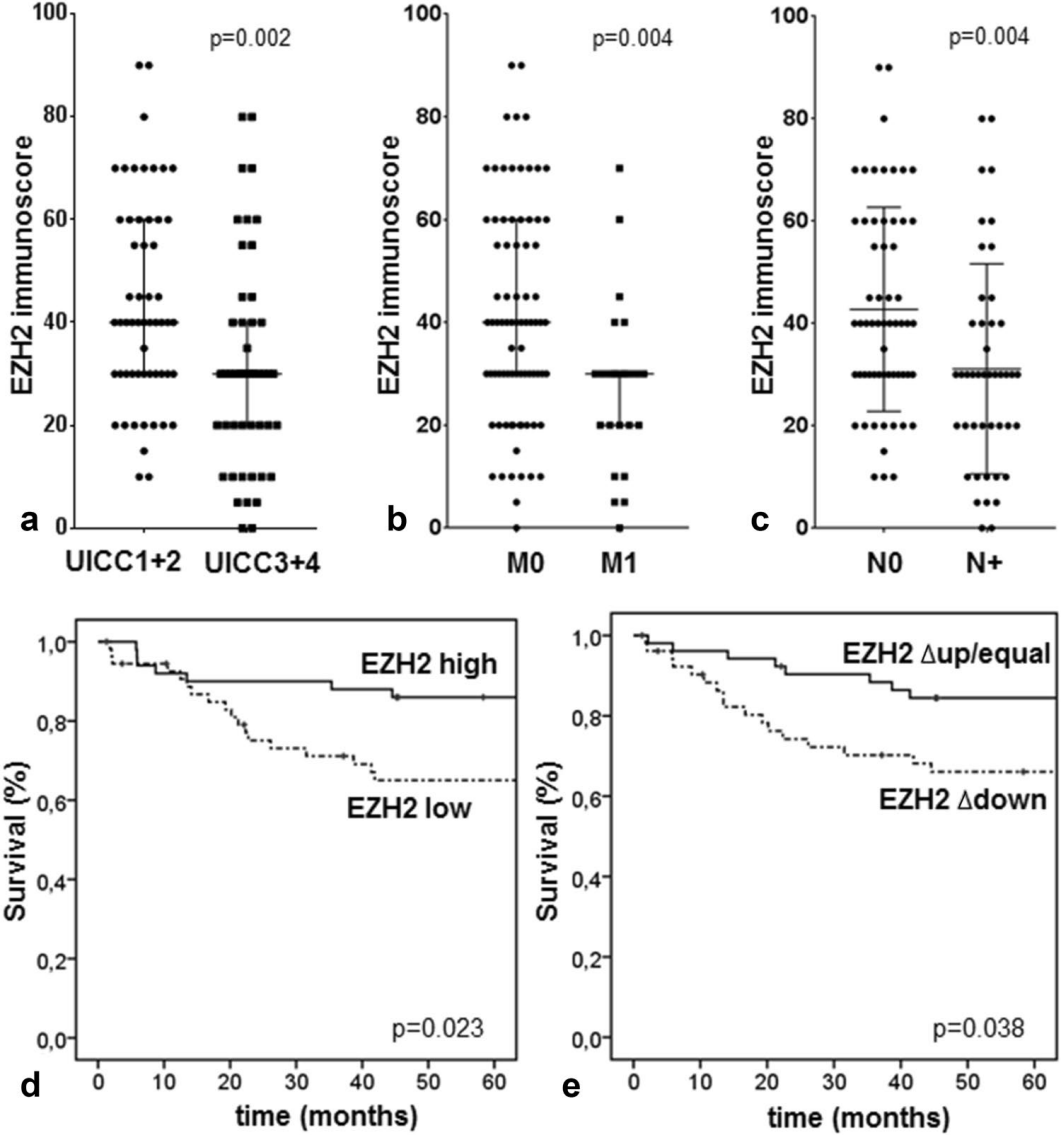
Correlation of EZH2 expression with clinicopathologic features. **a**–**c** A loss of EZH2 immunoscore at the invasion front is highly correlated to UICC stages 3 + 4, distant metastases, and lymph node metastases. Cloud diagrams show the median ± range. Kaplan–Meier plot demonstrates that lower EZH2 expression (**d**) and the difference between the corresponding EZH2 immunoscores in the center and at the invasive front (Δcenter–invasion front) (**e**) correlate significantly with worse prognosis of patients

Even though EZH2 expression in the tumor center did not correlate with any of the clinical data, EZH2 expression at the tumor invasion front was significantly correlated with poor clinical outcome. In general, lower EZH2 expression could be linked to higher tumor aggressiveness (Table 2) as advanced tumors (UICC III and IV, or tumors with lymph node or distant metastases) showed lower EZH2 expression (Fig. 3a–c). Indeed, 10 of 42 patients (23.8%) with an EZH2 immunoscore of < 30 died of disease, whereas only 3 of 42 patients (7.1%) with tumors highly expressing EZH2 died of disease. Low EZH2 expression was significantly correlated with an unfavorable prognosis (*p* = 0.023; Fig. 3d). In the Univariate Cox regression model, we found an approximately 0.4-fold risk of dying of disease for patients who had high EZH2 expressing tumors (Table 2; cut-off score at 30). Next, we estimated the difference between the corresponding EZH2 immunoscores in the center and at the invasive front (Δcenter–invasion front). Tumors that showed a loss of EZH2 at the invasion front (i.e., a reduction of more than ten) had a worse prognosis (*p* = 0.038; Fig. 3e). In contrast, tumors that had an equal EZH2 expression in both the areas or even an increased EZH2 immunoscore at the invasion front had a more favorable prognosis. In the Univariate Cox regression model, a reduction in the EZH2 immunoscore of > 10 was associated with a 2.1-fold increased risk of dying of disease (Table 2). The EZH2 Δcenter–invasion front score reached significance in the Multivariate Cox analysis suggesting an independent prognostic value in CRC (Table 2).

### Modeling EZH2 low expression tumors in CAM xenografts

**Fig. 4:**
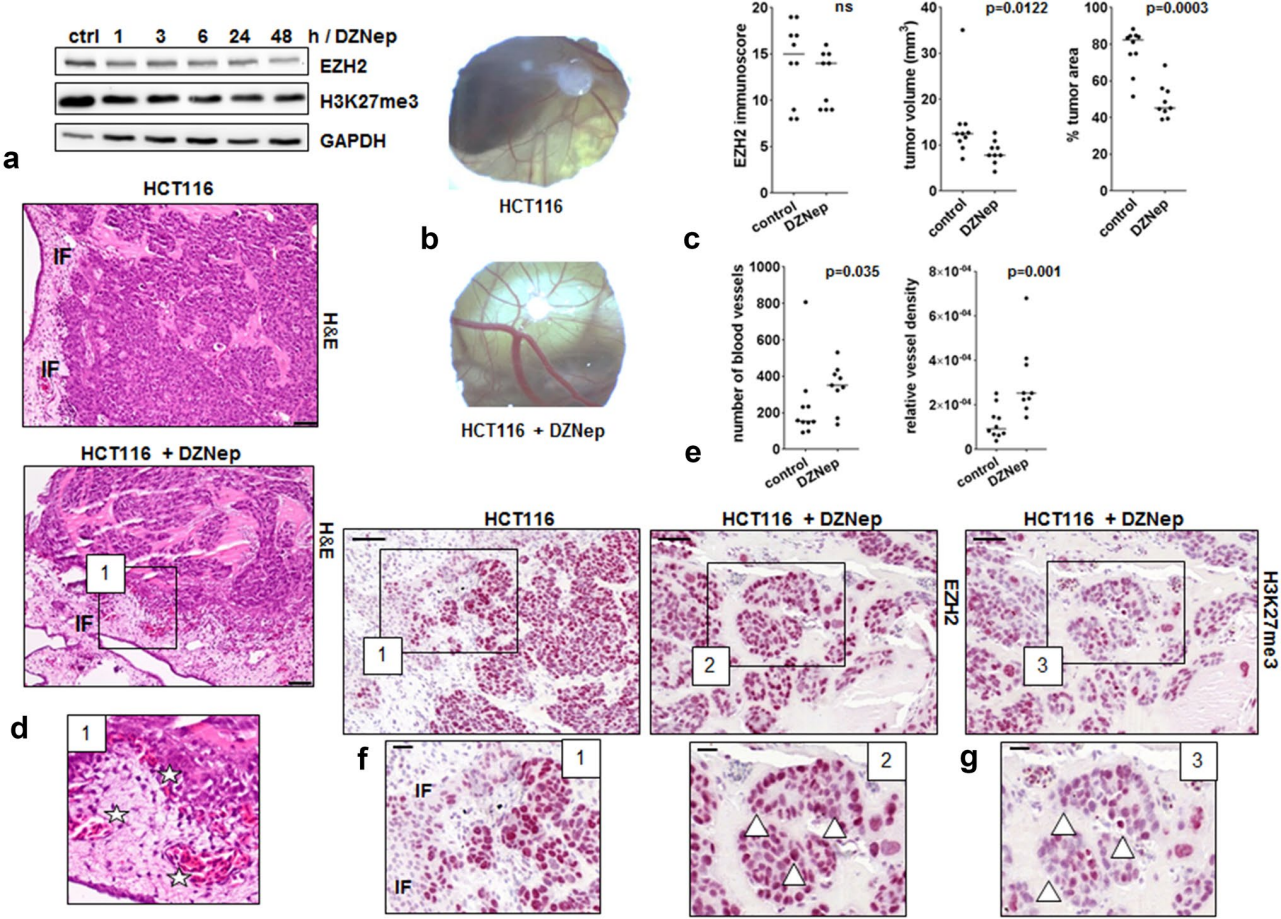
Evaluation of in vivo staining pattern using the CAM assay. **a** HCT116 cells were treated with 5 µM DZNep and time-dependent effects on the expression of EZH2 were analyzed by Western Blot. **b** Exemplary in ovo microtumor images. **c** EZH2 immunoscore as determined from CAM assay micro-xenografts of untreated (*n* = 10) and DZNep-treated (*n* = 9) HCT116 cells; tumor volume of CAM assay microtumors of untreated (*n* = 10) and DZNep-treated (*n* = 9) HCT116 cells; tumor cell area as obtained from analysis of HE stainings of untreated (*n* = 10) and DZNep pre-treated (*n* = 9) HCT116 microtumors. **d** Exemplary H&E stainings of CAM assay microtumors of untreated and DZNep-treated HCT116 cells; *IF* invasion front; magnification × 20, scale bar 50 µm. **d.1** Manually enlarged image of exemplary H&E staining, stars: chicken vessels with nucleated erythrocytes. **e** Number of blood vessels as determined by counting the number of blood vessels in the total area of untreated (*n* = 10) and DZNep-treated (*n* = 9) HCT116 microtumors; vessel density as calculated from area of blood vessels in the total area of microtumors of untreated (*n* = 10) and DZNep-treated (*n* = 9) HCT116 cells. **f** Exemplary EZH2 stainings of CAM assay microtumors of untreated and DZNep-treated HCT116 cells; magnification × 20, scale bar 50 µm. **f.1**, **f.2** Manually enlarged images of exemplary EZH2 stainings; triangles showing different EZH2 intensity; magnification × 40, scale bar 20 µm. **g** Exemplary H3K27me3 staining of CAM assay microtumors of DZNep-treated HCT116 cells, magnification × 20, scale bar 50 µm. **g.1** Manually enlarged images of exemplary H3K27me3 staining with different staining intensity (triangle), magnification × 40, scale bar 20 µm

We aimed to simulate the observed EZH2 loss at the invasion front in human tumors by treating HCT116 colorectal cancer cells with the EZH2 inhibitor DZNep and transplanted 10^6^ of these cells onto the CAM of 9-day old chicken embryos. IC_30_ range has been determined by Crystal Violet assay for suitable treatment of HCT116 cells (Online Resource 5a). Western Blot analysis showed that EZH2 protein levels slightly decreased after DZNep treatment (Fig. 4a). In ovo CAM, xenografts are exemplarily shown in Fig. 4b. In our study, we could observe that DZNep treatment also led to a slight reduction in EZH2 levels in CAM tumors (*n* = 9) when compared to untreated HCT116 control (*n* = 10) CAM microtumors (Fig. 4c, EZH2 immunoscore). In general, DZNep CAM microtumors were smaller (Fig. 4c, tumor volume) and exhibited a lower number of cells growing in clusters with large cell-free Matrigel areas, which would be expected due to the well-known stop of cell proliferation caused by DZNep treatment (Fig. 4c, Online Resource 4b) (Sha et al. 2015). Despite their cell cycle arrest, we observed that DZNep-treated tumor cells showed a highly invasive growth into the CAM (Fig. 4d) and their tumors displayed a higher grade of vascularization. A significantly greater number of vessels as well as a higher vessel density per graft area (Fig. 4e) in DZNep-treated CAM tumors demonstrated a pronounced aggressive phenotype when EZH2 is lost. A reduction of EZH2 at the invasion front could be verified in both treated and untreated HCT116 CAM xenografts corroborating our clinical data (Fig. 4f.1). Additionally, a very high heterogeneity regarding the intensity of EZH2 and H3K27me3 code staining could be detected in the tumors formed by DZNep-treated HCT116 cells (Fig. 4f.2, g). The higher vessel density is exemplarily displayed by HE staining in Fig. 4d.1.

### Tissue Studio software (TSS)

The analysis of the semi-automated software was compared to the manual one using the above-mentioned scoring technique. Evaluating a total number of 766 punches, the interobserver correlation between the TSS and two experts (JB, AVR) was highly significant for EZH2 (*r*_s_ = 0.289; *p* < 0.001) and H3K27me3 (*r*_s_ = 0.796; *p* < 0.001).

**Fig. 5:**
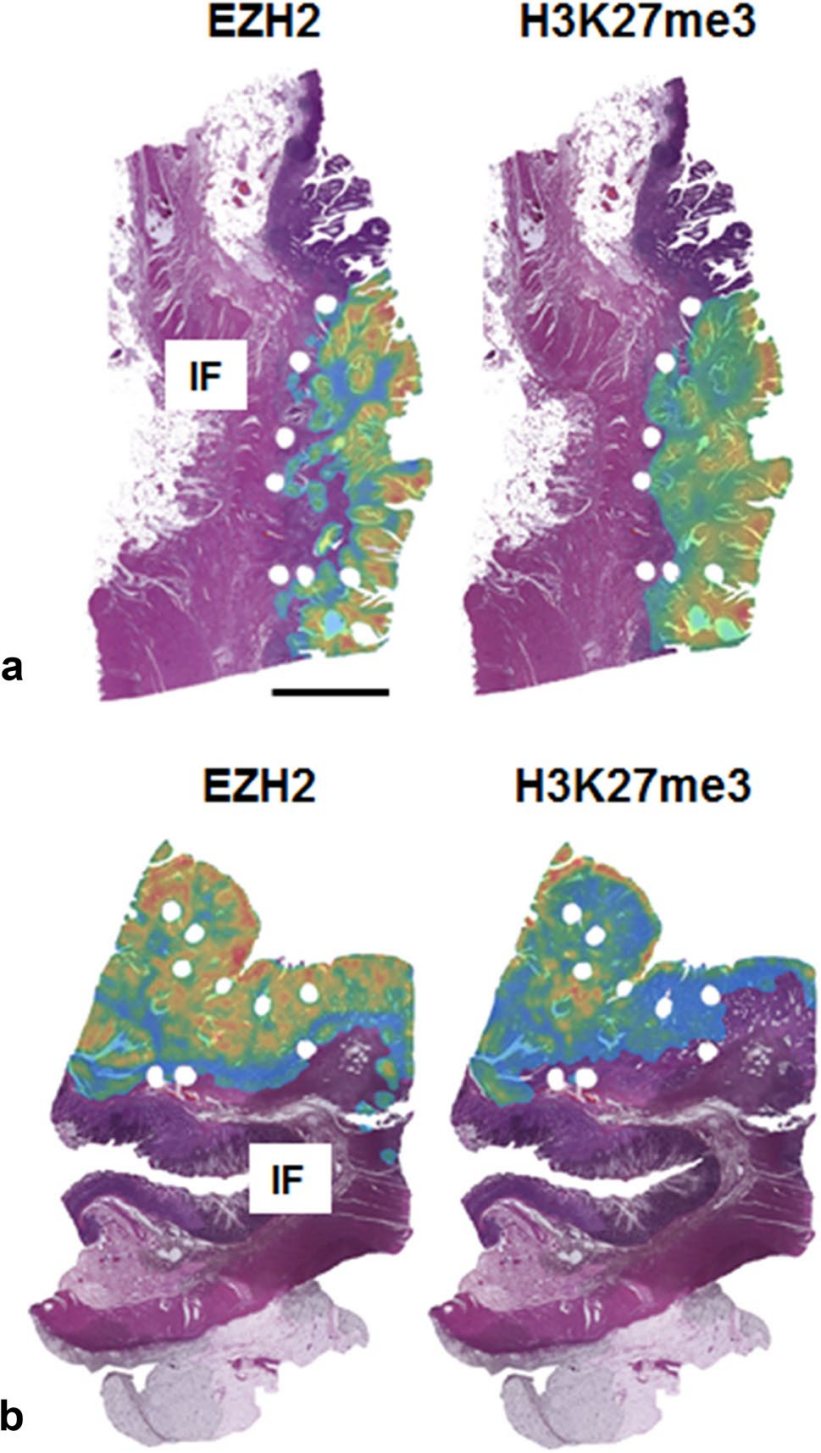
Heatmap construction. **a**, **b** Two examples of whole H&E-stained tissue slices with heatmap overlay showing the density of EZH2/H3K27me3 positive cells: low density areas are given in green, high density areas are marked in red. The invasion front (IF) is predominantly colored in green. Scale bar 5000 µm

To validate the TMA data for the tumor center, we selected eight whole tissue sections and compared the results of the manual evaluation of EZH2 immunostainings with the results obtained from core punches represented on the TMA. We were able to show a high concordance between immunostaining scores obtained from core punch and their corresponding whole tissue sections in seven out of eight cases. With the help of TSS, we constructed heatmaps that show the density of EZH2 and H3K27me3 positive cells by decoding it into different colors (Figs. 1, 5). Here, the software confirmed the results of the manual analysis demonstrating a remarkable loss of EZH2 and the corresponding repressive H3K27me3 code expression at the tumor invasion front compared to the tumor center.

## Discussion

EZH2, a component of the polycomb repressive complex 2, is involved in stable transcriptional repression during embryogenesis and under pathological conditions such as cancer. It is an important epigenetic regulator for genes that are involved in differentiation, proliferation, and stem cell renewal (Kim and Roberts 2016). In cancer, it has been reported that EZH2 seems to play a dual role as either an oncogene or tumor suppressor; however, the molecular mechanisms behind this antagonistic duality are not well understood (Margueron and Reinberg 2011). For this reason, it is not surprising that reports about the prognostic significance of EZH2 in cancer are highly divergent. EZH2 overexpression has been associated with aggressive tumor growth in breast cancer, in endometrial carcinomas, and melanoma as well as in prostate cancer (Bachmann et al. 2006). In these tumor entities, a high EZH2 expression has been linked to advanced disease and worse prognosis. In contrast, 2 recent meta-analyses concerning EZH2 expression in CRC tumors, one including 8 studies and more than 1059 CRC patients, while the other one comprised 10 studies with 1461 CRC patients, showed that EZH2 overexpression indicates a better prognosis in CRC and that EZH2 is not an indicator of poor prognosis, respectively (Vilorio-Marqués et al. 2017). With our study, we support the latter findings: there is no prognostic potential for EZH2 in the center of colon tumors. Nevertheless, loss of EZH2 at the tumor invasion front seems to be correlated with a more aggressive phenotype of cancer cells in colon cancer patients. By carefully evaluating the opposing data in the literature about the prognostic value of EZH2 in colon cancer, we recognized marked differences in the study designs that may contribute to these controversies. First, most studies did not distinguish between patients with colon and rectal cancer (Benard et al. 2014; Mimori et al. 2005; Takawa et al. 2011). Indeed, Fluge et al. found strong differences when analyzing colon and rectal tumors separately and reported a significant correlation of low EZH2 expression to relapse-free survival only in colon, but not in rectal cancer (Fluge et al. 2009). Thus, mixed groups of colon and rectal cancer can strongly influence the prognostic value of EZH2 expression. For this reason, we only analyzed colon tumors. Second, there are no studies considering a possible heterogeneity of EZH2 expression in the center and at the invasion front of a tumor. Interestingly, we found a significant decrease in EZH2 staining at the invasion front of colon tumors in our study. One would expect that genes that are upregulated by the low H3K27me3 code are different in normal cells and in cells at the tumor invasion front. In both areas, we see low EZH2/H3K27me3 code expression; nevertheless the functional consequences have to be completely different from each other. Moreover, the lack of a significant correlation between EZH2 and its repressive code might lead to the conclusion that there are also H3K27me3 independent functions of EZH2.

In addition to the well-known general involvement of EZH2 in cell cycle regulation, which especially affects cells with high mitotic activity (Simon and Lange 2008), it was also found that a loss of EZH2 in ovarian cancer stem cells is correlated with growth inhibition. Since cancer stem cells are thought to be found at the tumor invasion front, proliferation stop seems to be a necessary prerequisite for cellular plasticity with subsequent changes in cell phenotype that enable migration and invasion into the surrounding tissue. Additionally, it should be mentioned that EZH2 expression was reported to facilitate transformation by blocking lineage specification (Ezhkova et al. 2009). Interestingly, EZH2 also represses the enterocyte differentiation program in the intestine (Benoit et al. 2012). Moreover, it is suggested that EZH2 specifically suppresses transcriptional programs associated with alternative phenotypes. Also the finding that a complete elimination of EZH2 is impossible in certain tumor cell lines due to gene essentiality shows that fate control by EZH2 is strongly cell type specific (Grassian et al. 2015). Loss of EZH2 and mitotic activity at the invasion front of colon cancer seems to play an important role for the acquisition of mesenchymal characteristics that are required for the migration and invasion of disseminating tumor and cancer stem cells (Palmqvist et al. 1998, 1999, 2000; Jung et al. 2001; Jie et al. 2007). This leads to the conclusion that a reduction or complete loss of EZH2 expression resulting in a stop of proliferation represents a promoting factor associated with the dissemination of tumor cells and the formation of metastases. Indeed, we found a correlation between EZH2 and survival of patients only when considering its expression at the invasion front. In this regard, we detected more lymph node and distant metastases, and higher UICC stages for patients with a lower EZH2 expression at the invasion front. Interestingly the gradient in EZH2 expression reached significance in the Multivariate Cox’s analysis suggesting that the evaluation of a decrease in EZH2 at invasion front is an independent prognostic factor in CRC. This is promising since the detection of a difference in EZH2 expression should be independent of the scoring system used in different labs. There was no significant correlation between the budding score or the MMRP status and EZH2 expression neither in the center nor at the invasion front. In a study of Joensuu et al., they found a positive correlation between microsatellite unstable tumors and high EZH2 expression (Joensuu et al. 2015). Although it is well known that microsatellite unstable tumors have a better prognosis, we could not confirm this in our tumor group. The reason might be the composition of our tumor group that consists of a mixture of stage I–IV tumors, and thus the prognostic validity of the link between M1 CRC patients with proximal tumors and worse prognosis is masked (Merkel et al. 2018).

It is already well accepted that tumors with a more aggressive invasion front harm the patients more severely and influence their survival. The analysis of different tumor areas in the various studies present in the current literature could explain their strong discrepancies. It is possible that depending on which tumor site is being analyzed, the effect of the invasion front is represented in the study results, while in other cases more luminal parts are examined and, therefore, the characteristics of the invasion front are (largely) neglected. As proposed by Wassermann et al. for p16INK4A also for EZH2, there might only be a small subpopulation of tumor cells that determines the prognosis and that differs from the rest of the tumor (Wassermann et al. 2009).

Since CRISPR-Cas ko for EZH2 in HC116 cells was not successful (data not shown) in an attempt to mimic EZH2 status of CRC tumors at the invasion front, we treated HCT116 colon tumor cells with a common EZH2 inhibitor (DZNep) that is known to decrease the activity and protein level of EZH2 (Tan et al. 2007; Sun et al. 2009). We applied the in ovo CAM xenograft model to compare the growth of control and DZNep-treated HCT116 cells. Remarkably, DZNep-treated HCT116 cells formed microtumors showing high heterogeneity for EZH2 staining and expression of the corresponding H3K27me3 code and a distinct decrease in the immunoscores at the tumor invasion front. Moreover, we were able to confirm the drug-induced growth arrest caused by DZNep treatment for the first time in vivo, since tumors formed by DZNep-treated HCT116 cells were only loosely packed and showed clearly reduced cell numbers. As anticipated in our model system, tumors of DZNep-treated cells displayed a more aggressive phenotype. They showed a pronounced invasive behavior with many cell clusters and buds infiltrating the CAM. Moreover, a high vessel density could be observed. These results underline our findings in patients and the fact that EZH2 loss leads to a growth inhibition, which represents an important mechanism for disseminating tumor cells. As already mentioned above, a proliferation stop at the invasion front of colon cancer has already been reported in various other studies (Palmqvist et al. 1998, 1999, 2000). Moreover, it has been shown that the massive upregulation of the cell cycle inhibitor p16(INK4a) at the tumor invasion front was strongly associated with a decreased Ki67 proliferation index (Jie et al. 2007; Jung et al. 2001). In conclusion, our findings give further evidence for EZH2 having a tumor suppressor role in disseminating tumor cells, which adds a new aspect of bifunctional nature to this chromatin-modifying enzyme for determining tumor heterogeneity.

Further reasons for the divergent data in the literature are the various scoring systems and different cutoff values used for the quantification of EZH2 expression that prevent a reliable comparison of available studies, even though they are all focusing on CRC. For example, Mimori et al. and Liu et al. used qRT-PCR, while Fluge et al., Takawa et al., and Meng et al. used IHC and Benard et al. combined both techniques in their studies, hence applying different quantification criteria and grades of sensitivity for the determination of EZH2 expression (Benard et al. 2014; Fluge et al. 2009; Liu et al. 2015; Mimori et al. 2005; Takawa et al. 2011; Meng et al. 2014). Even within only IHC-based studies, the definition of high expression ranged from > 30 to > 50% EZH2 positive cells or was not clearly specified in the reports, while the intensity of a positive staining was not considered at all (Takawa et al. 2011). Only Fluge et al. applied a staining index that included the intensity with values ranging from 1 to 3 (Fluge et al. 2009). Yamamoto et al. used a dichotomous expression variable with 40% as a cutoff between low- and high-expressing EZH2 tumors (Yamamoto et al. 2017). Thus, a reproducible and time efficient method for EZH2 scoring could help to standardize the measurement of EZH2 staining patterns and to establish EZH2 as a reliable biomarker in colon cancer. So far, except for a single report, the H3K27me3 code has not been correlated with EZH2 expression in these studies (Benard et al. 2014). We successfully applied our novel semi-automated software tool for detection of nuclear EZH2 expression. The strong interobserver correlation shows that Definiens TSS is a suitable tool for the analysis of big data sets such as TMAs. Since the training of the software is very time consuming, especially for inexperienced researchers, its use is especially recommended for the analysis of big data sets. Applying the software can save time and also reduce the risk of errors, especially when analyzing TMAs with a high density of punches. As the threshold of the intensity cannot be changed in the same run, the staining quality should be equal between the different specimens; a requirement that is fulfilled today using automatic staining procedures. The visualization of the results in that heatmaps is an excellent software feature.

In conclusion, we suggest that heterogeneity of EZH2 expression concerning tumor center and invasion front as well as different scoring and cutoff values can most likely explain the controversial literature data concerning the prognostic value of EZH2. We further propose that, independent of the used scoring system, a remarkable loss of EZH2 from tumor center to the invasion front could be a suitable prognosticator of worse prognosis in daily routine. Finally, we recommend that epigenetic therapies with EZH2 inhibitors should be carefully evaluated for each specific tumor type, since alterations in cell differentiation might lead to unfavorable results.

## Electronic supplementary material

Below is the link to the electronic supplementary material.

Supplementary material 1 (DOCX 13 kb)

**Online Resource 1 Example of budding at the invasion front of a G2 colon cancer with** (**a**) low grade tumor-budding (4 buds circled blue within 0,78 mm^2^ representing a hot spot of tumor budding and (**b**) intermediate grade tumor-budding (7 buds circled blue within 0,78 mm^2^ representing a hot spot of tumor budding) and (**c**) of a G3 colon cancer with high grade tumor-budding (20 buds circled blue within 0,78 mm^2^ representing a hot spot of tumor budding). Classification was done regarding Lugli et al. (2017) - Detected by eye on digital slides in 10-20x in standardized area annotation in the Viewer software CaseViewer Ver.2.0. Analyzed standard area was 0,787 mm^2^. Digital slide specifications: Scanner Pannoramic Flash 250, Software: 1.15.0.50, scanned with Plan-Apochromat 20x, Camera type: CIS VCC F52U25CL, solution: micrometer/pixel: 0.221 (TIFF 3151 kb)

**Online Resource 2 Images of whole colon tumor slices (x5 magnification) showing the area of punches for the TMA** There is no or only little heterogeneity in the surrounding area of punches demonstrating that TMAs are representative for the whole tissue slice (TIFF 3991 kb)

**Online Resource 3 Examples of different intensities for the EZH2 and H3K27me3 code score** (40x magnification), arrows show exemplary cells with corresponding intensity score (TIFF 3697 kb)

**Online Resource 4 Survival analyses for clinicopathological features in patients with colon carcinoma (5-year cancer-related survival rates)** (**a**) Males 67.2%, females 84.0%, p = 0.059; (**b**) UICC I 100%, UICC II 97.1%, UICC III 81.8%, UICC IV 20.0%, p < 0.001; (**c**) pT1,2 100%, pT3 73.4%, pT4 54.5%, p < 0.001; (**d**) pN0 97.9%, pN1,2 48.9%, p < 0.001; (**e**) M0 93.4%, M1 20.0%, p < 0.001; (f) low/intermediate 81.6%, high budding 50%, p = 0.013; (**g**) low grade 80.1%, high grade 66.8%, p = 0.401 (TIFF 2202 kb)

**Online Resource 5 Crystal Violet assay and Flow cytometric analysis of cell cycle distribution** (**a**) Treatment of HCT116 cells with different concentrations of DZNep (0.25 – 100 µM). Cell viability was assessed by crystal violet assay after 48 h of incubation and expressed as percentage of respective DMSO controls. (**b**) Cell populations in the cell cycle phases G1, S and G2/M as well as the apoptotic fraction (sub-G1) of control and DZNep treated HCT116 cells after 24 h of incubation as determined by propidium iodide staining. Values represent means of two replicates. (TIFF 2248 kb)

## References

[CR1] Bachmann IM, Halvorsen OJ, Collett K et al (2006) EZH2 expression is associated with high proliferation rate and aggressive tumor subgroups in cutaneous melanoma and cancers of the endometrium, prostate, and breast. J Clin Oncol 24:268–273. 10.1200/JCO.2005.01.5180 16330673 10.1200/JCO.2005.01.5180

[CR2] Benard A, Goossens-Beumer IJ, van Hoesel AQ et al (2014) Prognostic value of polycomb proteins EZH2, BMI1 and SUZ12 and histone modification H3K27me3 in colorectal cancer. PLoS One 9:e108265. 10.1371/journal.pone.0108265 25243792 10.1371/journal.pone.0108265 PMC4171510

[CR3] Benoit YD, Lepage MB, Khalfaoui T et al (2012) Polycomb repressive complex 2 impedes intestinal cell terminal differentiation. J Cell Sci 125:3454–3463. 10.1242/jcs.102061 22467857 10.1242/jcs.102061 PMC3516381

[CR4] Chen JF, Luo X, Xiang LS et al (2016) EZH2 promotes colorectal cancer stem-like cell expansion by activating p21cip1-Wnt/β-catenin signaling. Oncotarget 7:41540–41558. 10.18632/oncotarget.9236 27172794 10.18632/oncotarget.9236 PMC5173077

[CR5] Crea F, Fornaro L, Bocci G et al (2012) EZH2 inhibition: targeting the crossroad of tumor invasion and angiogenesis. Cancer Metastasis Rev 31:753–761. 10.1007/s10555-012-9387-3 22711031 10.1007/s10555-012-9387-3

[CR6] Ezhkova E, Pasolli HA, Parker JS et al (2009) Ezh2 orchestrates gene expression for the stepwise differentiation of tissue-specific stem cells. Cell 136:1122–1135. 10.1016/j.cell.2008.12.043 19303854 10.1016/j.cell.2008.12.043 PMC2716120

[CR7] Fluge Ø, Gravdal K, Carlsen E et al (2009) Expression of EZH2 and Ki-67 in colorectal cancer and associations with treatment response and prognosis. Br J Cancer 101:1282–1289. 10.1038/sj.bjc.6605333 19773751 10.1038/sj.bjc.6605333 PMC2768450

[CR8] Fussbroich B, Wagener N, Macher-Goeppinger S et al (2011) EZH2 depletion blocks the proliferation of colon cancer cells. PLoS One 6:e21651. 10.1371/journal.pone.0021651 21765901 10.1371/journal.pone.0021651 PMC3135584

[CR9] Grassian AR, Scales TME, Knutson SK et al (2015) A medium-throughput single cell CRISPR-Cas9 assay to assess gene essentiality. Biol Proc Online 17:15. 10.1186/s12575-015-0028-4 10.1186/s12575-015-0028-4 PMC4647477 26578851

[CR10] He SB, Zhou H, Zhou J et al (2014) Inhibition of EZH2 expression is associated with the proliferation, apoptosis, and migration of SW620 colorectal cancer cells in vitro. Exp Biol Med (Maywood) 240:458–466. 10.1177/1535370214542215 25005166 10.1177/1535370214542215 PMC4935379

[CR11] Jie G, Zhixiang S, Lei S, Hesheng L, Xiaojun T (2007) Relationship between expression and methylation status of p16INK4a and the proliferative activity of different areas’ tumour cells in human colorectal cancer. Int J Clin Pract 61:1523–1529. 10.1111/j.1742-1241.2006.01033.x 17537196 10.1111/j.1742-1241.2006.01033.x

[CR12] Joensuu EI, Nieminen TT, Lotsari JE, Pavicic W, Abdel-Rahman WM, Peltomäki P (2015) Methyltransferase expression and tumor suppressor gene methylation in sporadic and familial colorectal cancer. Genes Chromosomes Cancer 54(12):776–787. 10.1002/gcc.22289 26305882 10.1002/gcc.22289

[CR13] Jung A, Schrauder M, Oswald U et al (2001) The invasion front of human colorectal adenocarcinomas shows co-localization of nuclear beta-catenin, cyclin D1, and p16INK4A and is a region of low proliferation. Am J Pathol 159:1613–1617 11696421 10.1016/s0002-9440(10)63007-6 PMC1867079

[CR14] Kim KH, Roberts CW (2016) Targeting EZH2 in cancer. Nat Med 22:128–134. 10.1038/nm.4036 26845405 10.1038/nm.4036 PMC4918227

[CR15] Kuzmichev A, Nishioka K, Erdjument-Bromage H, Tempst P, Reinberg D (2002) Histone methyltransferase activity associated with a human multiprotein complex containing the enhancer of zeste protein. Genes Dev 16:2893–2905. 10.1101/gad.1035902 12435631 10.1101/gad.1035902 PMC187479

[CR17] Liu YL, Gao X, Jiang Y et al (2015) Expression and clinicopathological significance of EED, SUZ12 and EZH2 mRNA in colorectal cancer. J Cancer Res Clin Oncol 141:661–669. 10.1007/s00432-014-1854-5 25326896 10.1007/s00432-014-1854-5 PMC11823763

[CR18] Lugli A, Kirsch R, Ajioka Y et al (2017) Recommendations for reporting tumor budding in colorectal cancer based on the International Tumor Budding Consensus Conference (ITBCC) 2016. Mod Pathol 30(9):1299–1311. 10.1038/modpathol.2017.46 28548122 10.1038/modpathol.2017.46

[CR19] Margueron R, Reinberg D (2011) The Polycomb complex PRC2 and its mark in life. Nature 469:343–349. 10.1038/nature09784 21248841 10.1038/nature09784 PMC3760771

[CR20] Meng X, Huang Z, Wang R et al (2014) The prognostic role of EZH2 expression in rectal cancer patients treated with neoadjuvant chemoradiotherapy. Radiat Oncol 9:188. 10.1186/1748-717X-9-188 25159232 10.1186/1748-717X-9-188 PMC4163172

[CR21] Merkel S, Schellerer VS, Wein A et al (2018) The influence of tumour site on prognosis in metastatic colorectal carcinomas with primary tumour resection. Int J Colorectal Dis. 10.1007/s00384-018-3098-3 29915904 10.1007/s00384-018-3098-3

[CR22] Mimori K, Ogawa K, Okamoto M, Sudo T, Inoue H, Mori M (2005) Clinical significance of enhancer of zeste homolog 2 expression in colorectal cancer cases. Eur J Surg Oncol 31:376–380. 10.1016/j.ejso.2004.11.001 15837043 10.1016/j.ejso.2004.11.001

[CR23] Muenzner JK, Kunze P, Lindner P (2018) Generation and characterization of hepatocellular carcinoma cell lines with enhanced cancer stem cell potential. J Cell Mol Med. 10.1111/jcmm.13911 30280520 10.1111/jcmm.13911 PMC6237557

[CR24] Nolte S, Zlobec I, Lugli A et al (2017) Construction and analysis of tissue microarrays in the era of digital pathology: a pilot study targeting CDX1 and CDX2 in a colon cancer cohort of 612 patients. J Pathol Clin Res 3:58–70. 10.1002/cjp2.62 28138402 10.1002/cjp2.62 PMC5259563

[CR25] Palmqvist R, Oberg A, Bergström C, Rutegård JN, Zackrisson B, Stenling R (1998) Systematic heterogeneity and prognostic significance of cell proliferation in colorectal cancer. Br J Cancer 77:917–925 9528835 10.1038/bjc.1998.152 PMC2150107

[CR26] Palmqvist R, Sellberg P, Oberg A, Tavelin B, Rutegård JN, Stenling R (1999) Low tumour cell proliferation at the invasive margin is associated with a poor prognosis in Dukes’ stage B colorectal cancers. Br J Cancer 79:577–581. 10.1038/sj.bjc.6690091 10027333 10.1038/sj.bjc.6690091 PMC2362413

[CR27] Palmqvist R, Rutegârd JN, Bozoky B, Landberg G, Stenling R (2000) Human colorectal cancers with an intact p16/cyclin D1/pRb pathway have up-regulated p16 expression and decreased proliferation in small invasive tumor clusters. Am J Pathol 157:1947–1953. 10.1016/S0002-9440(10)64833-X 11106567 10.1016/S0002-9440(10)64833-X PMC3277300

[CR28] Ribatti D (2017) The chick embryo chorioallantoic membrane (CAM) assay. Reprod Toxicol 70:97–101. 10.1016/j.reprotox.2016.11.004 27832950 10.1016/j.reprotox.2016.11.004

[CR29] Sha M, Mao G, Wang G, Chen Y, Wu X, Wang Z (2015) DZNep inhibits the proliferation of colon cancer HCT116 cells by inducing senescence and apoptosis. Acta Pharm Sin B 5:188–193. 10.1016/j.apsb.2015.01.011 26579445 10.1016/j.apsb.2015.01.011 PMC4629229

[CR30] Simon JA, Lange CA (2008) Roles of the EZH2 histone methyltransferase in cancer epigenetics. Mutat Res 647:9. 10.1016/j.mrfmmm.2008.07.010 10.1016/j.mrfmmm.2008.07.010 18723033

[CR31] Sun F, Chan E, Wu Z, Yang X, Marguez VE, Yu Q (2009) Combinatorial pharmacologic approaches target EZH2-mediated gene repression in breast cancer cells. Mol Cancer Ther 8:3191–3202. 10.1158/1535-7163.MCT-09-0479 19934278 10.1158/1535-7163.MCT-09-0479 PMC2794891

[CR32] Takawa M, Masuda K, Kunizaki M et al (2011) Validation of the histone methyltransferase EZH2 as a therapeutic target for various types of human cancer and as a prognostic marker. Cancer Sci 102:1298–1305. 10.1111/j.1349-7006.2011.01958.x 21539681 10.1111/j.1349-7006.2011.01958.x PMC11159278

[CR33] Tamagawa H, Oshima T, Numata M et al (2013) Global histone modification of H3K27 correlates with the outcomes in patients with metachronous liver metastasis of colorectal cancer. Eur J Surg Oncol 39:655–661. 10.1016/j.ejso.2013.02.023 23523318 10.1016/j.ejso.2013.02.023

[CR34] Tan J, Yang X, Zhuang L et al (2007) Pharmacologic disruption of Polycomb-repressive complex 2-mediated gene repression selectively induces apoptosis in cancer cells. Genes Dev 21:1050–1063. 10.1101/gad.1524107 17437993 10.1101/gad.1524107 PMC1855231

[CR35] Vilorio-Marqués L, Martín V, Diez-Tascón C et al (2017) The role of EZH2 in overall survival of colorectal cancer: a meta-analysis. Sci Rep 7:13806. 10.1038/s41598-017-13670-z 29061982 10.1038/s41598-017-13670-z PMC5653815

[CR36] Wassermann S, Scheel SK, Hiendlmeyer E et al (2009) p16INK4a is a beta-catenin target gene and indicates low survival in human colorectal tumors. Gastroenterology 136:196–205. 10.1053/j.gastro.2008.09.019 18951899 10.1053/j.gastro.2008.09.019

[CR37] Wei Y, Xia W, Zhang Z et al (2008) Loss of trimethylation at lysine 27 of histone H3 is a predictor of poor outcome in breast, ovarian, and pancreatic cancers. Mol Carcinog 47:701–706. 10.1002/mc.20413 18176935 10.1002/mc.20413 PMC2580832

[CR38] Yamamoto I, Nosho K, Kanno S et al (2017) EZH2 expression is a prognostic biomarker in patients with colorectal cancer treated with anti-EGFR therapeutics. Oncotarget 8:17810–17818. 10.18632/oncotarget.14863 28147317 10.18632/oncotarget.14863 PMC5392288

