# Supplementary material for: Loss of enhancer of zeste homologue 2 (EZH2) at tumor invasion front is correlated with higher aggressiveness in colorectal cancer cells
Source: J Cancer Res Clin Oncol. 2019 Jul 17;145(9):2227–40. doi: 10.1007/s00432-019-02977-1 (PMC6708512; doi:10.1007/s00432-019-02977-1)
Supplement: Supplementary file 1 — Supplementary material 1 (DOCX 13 kb) [file 432_2019_2977_MOESM1_ESM.docx]

| Antibody | Clone | Source | Dilution | Staining localization | Definition |
| --- | --- | --- | --- | --- | --- |
| MLH1 | ES05 Isotype: IgG1, kappa. | DAKO | 1:50 | nuclear | positive if > 10% of cells are nuclear stained |
| MSH6 | 44/MSH6 | BD Transduction Laboratories | 1:100 | nuclear | positive if > 10% of cells are nuclear stained |
| MSH2 | 760-4265  (G219-1129) | Ventana | None- ready to use | nuclear | positive if > 10% of cells are nuclear stained |
| PMS2 | EP51 | DAKO | 1:40 | nuclear | positive if > 10% of cells are nuclear stained |

Suppl. Table 1 Immunohistochemical procedures

Article title:

Loss of enhancer of zeste homologue 2 (EZH2) at tumor invasion front is correlated with higher aggressiveness in colorectal cancer cells

Journal name:

Journal of Cancer Research and Clinical Oncology

Author names:

Julian Böhm, Julienne Kathrin Muenzner, Aylin Caliskan, Benardina Ndreshkjana, Katharina Erlenbach-Wünsch, Susanne Merkel, Roland Croner, Tilman T. Rau, Carol Immanuel Geppert, Arndt Hartmann, Adriana Vial Roehe, Regine Schneider-Stock

Corresponding author:

Regine Schneider-Stock, PhD

Experimental Tumor Pathology

Institute of Pathology

University Hospital

Friedrich-Alexander-Universität Erlangen-Nürnberg

Universitaetsstrasse 22

91054 Erlangen

tel.: 0049-9131-8526069

fax: 0049-9131-8526197

e-mail: [regine.schneider-stock@uk-erlangen.de](mailto:regine.schneider-stock@uk-erlangen.de)
