# Supplementary figures and images for: Loss of enhancer of zeste homologue 2 (EZH2) at tumor invasion front is correlated with higher aggressiveness in colorectal cancer cells
Source: J Cancer Res Clin Oncol. 2019 Jul 17;145(9):2227–40. doi: 10.1007/s00432-019-02977-1 (PMC6708512; doi:10.1007/s00432-019-02977-1)

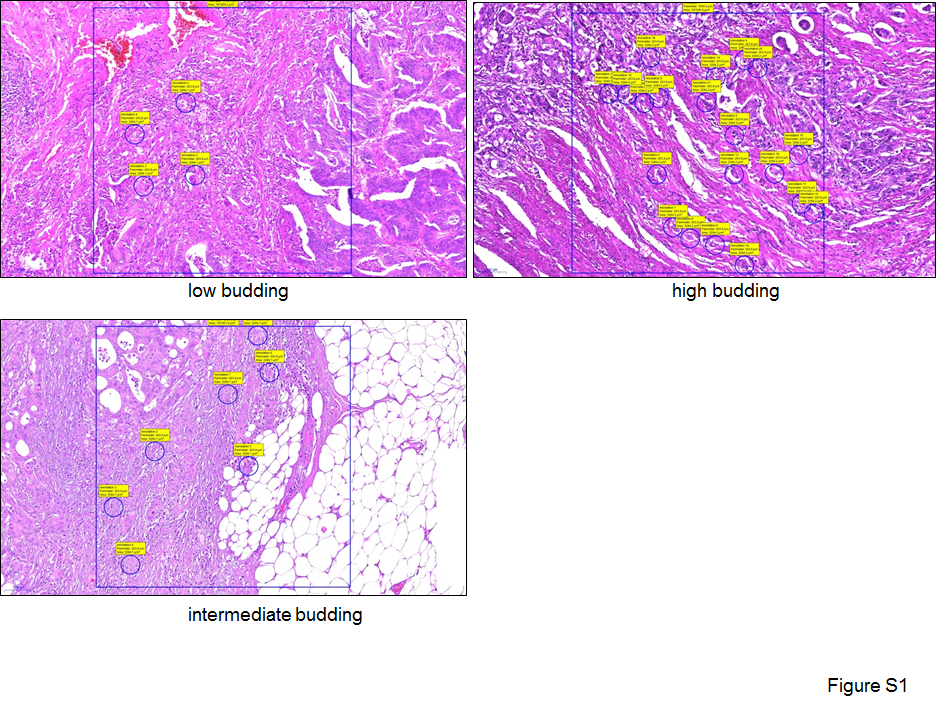

Supplement: Supplementary file 2 — Online Resource 1 Example of budding at the invasion front of a G2 colon cancer with (a) low grade tumor-budding (4 buds circled blue within 0,78 mm2 representing a hot spot of tumor budding and (b) intermediate grade tumor-budding (7 buds circled blue within 0,78 mm2 representing a hot spot of tumor budding) and (c) of a G3 colon cancer with high grade tumor-budding (20 buds circled blue within 0,78 mm2 representing a hot spot of tumor budding). Classification was done regarding Lugli et al. (2017) - Detected by eye on digital slides in 10-20x in standardized area annotation in the Viewer software CaseViewer Ver.2.0. Analyzed standard area was 0,787 mm2. Digital slide specifications: Scanner Pannoramic Flash 250, Software: 1.15.0.50, scanned with Plan-Apochromat 20x, Camera type: CIS VCC F52U25CL, solution: micrometer/pixel: 0.221 (TIFF 3151 kb) [file 432_2019_2977_MOESM2_ESM.tif]

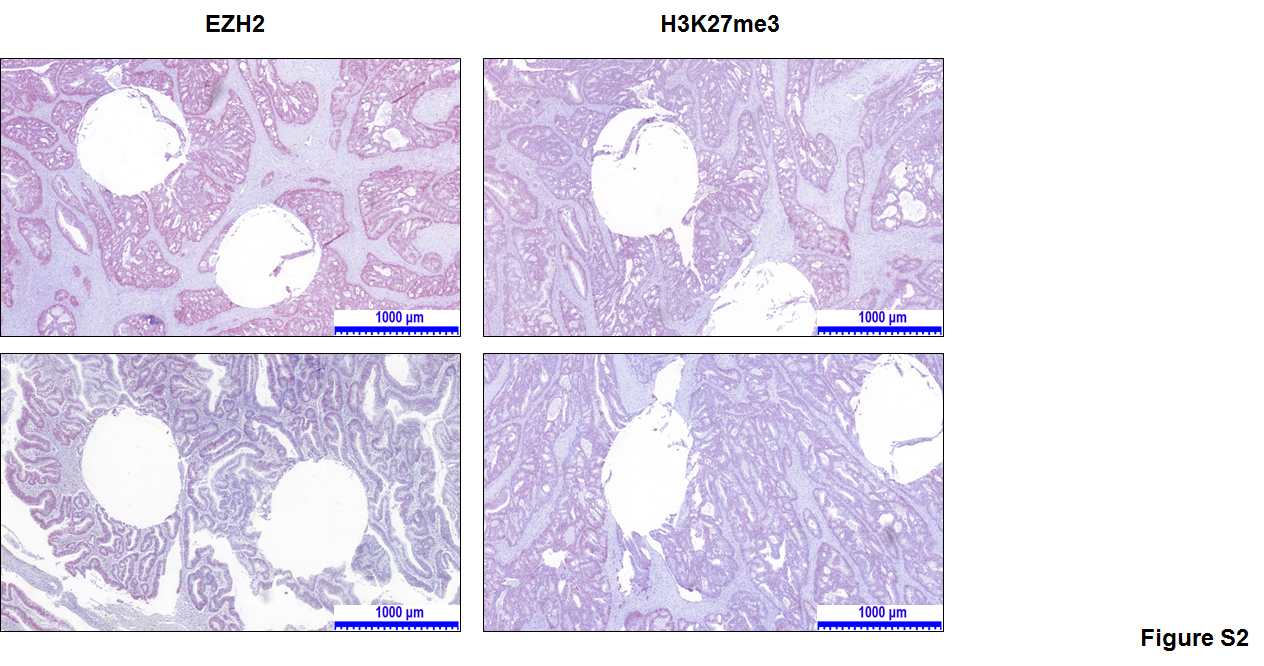

Supplement: Supplementary file 3 — Online Resource 2 Images of whole colon tumor slices (x5 magnification) showing the area of punches for the TMA There is no or only little heterogeneity in the surrounding area of punches demonstrating that TMAs are representative for the whole tissue slice (TIFF 3991 kb) [file 432_2019_2977_MOESM3_ESM.tif]

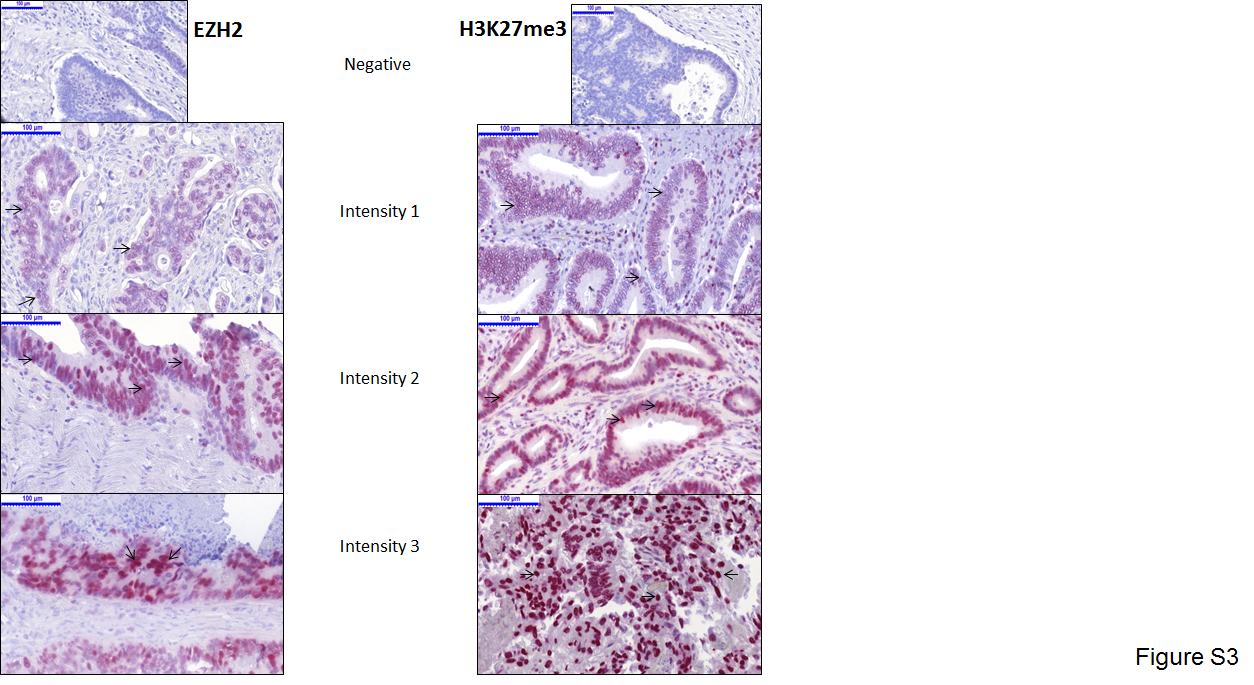

Supplement: Supplementary file 4 — Online Resource 3 Examples of different intensities for the EZH2 and H3K27me3 code score (40x magnification), arrows show exemplary cells with corresponding intensity score (TIFF 3697 kb) [file 432_2019_2977_MOESM4_ESM.tif]

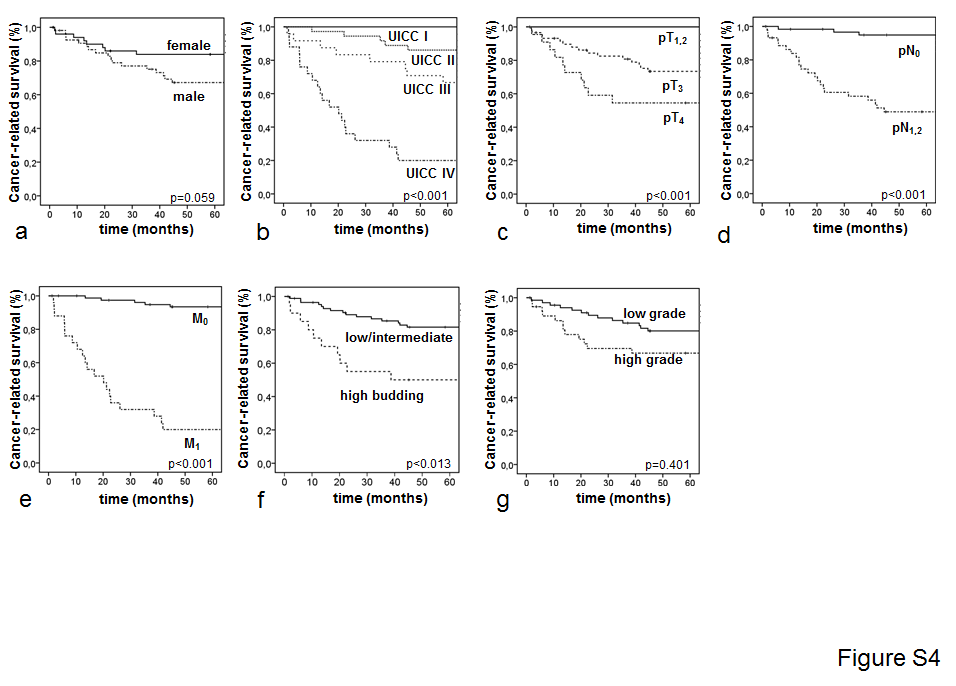

Supplement: Supplementary file 5 — Online Resource 4 Survival analyses for clinicopathological features in patients with colon carcinoma (5-year cancer-related survival rates) (a) Males 67.2%, females 84.0%, p = 0.059; (b) UICC I 100%, UICC II 97.1%, UICC III 81.8%, UICC IV 20.0%, p < 0.001; (c) pT1,2 100%, pT3 73.4%, pT4 54.5%, p < 0.001; (d) pN0 97.9%, pN1,2 48.9%, p < 0.001; (e) M0 93.4%, M1 20.0%, p < 0.001; (f) low/intermediate 81.6%, high budding 50%, p = 0.013; (g) low grade 80.1%, high grade 66.8%, p = 0.401 (TIFF 2202 kb) [file 432_2019_2977_MOESM5_ESM.tif]

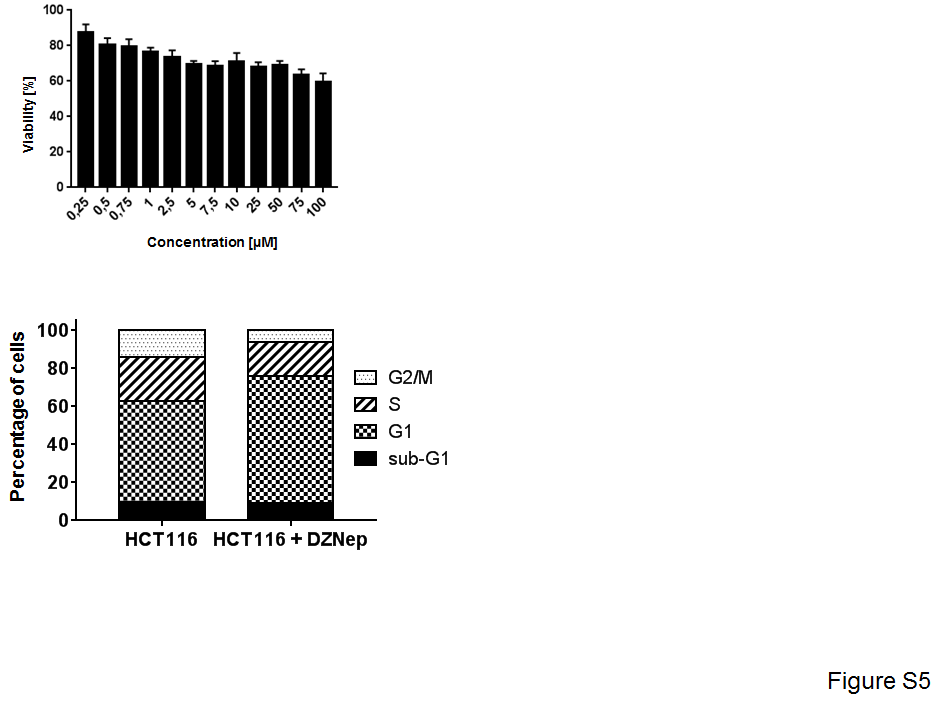

Supplement: Supplementary file 6 — Online Resource 5 Crystal Violet assay and Flow cytometric analysis of cell cycle distribution (a) Treatment of HCT116 cells with different concentrations of DZNep (0.25 – 100 µM). Cell viability was assessed by crystal violet assay after 48 h of incubation and expressed as percentage of respective DMSO controls. (b) Cell populations in the cell cycle phases G1, S and G2/M as well as the apoptotic fraction (sub-G1) of control and DZNep treated HCT116 cells after 24 h of incubation as determined by propidium iodide staining. Values represent means of two replicates. (TIFF 2248 kb) [file 432_2019_2977_MOESM6_ESM.tif]
